# Chronic kidney disease and cognitive decline: choroid plexus remodeling, glymphatic dysfunction, and Alzheimer biomarker interpretation

**DOI:** 10.1007/s10072-026-09362-0

**Published:** 2026-09-11

**Authors:** Lucas Maciel de Almeida Corrêa, Rebeca Oliveira da Silva, Luiggi Kevin Virgino Brandão, Yan Roberth Delmiro Silva, Éverton Victor Belmiro da Silva, Julia Tretow, Esther da Costa Gomes, Camilla Cristina Silva Fernandes, Gabriel Negreiros de Camargo Martins Moreira, Juliana Zanella

**Affiliations:** 1 Faculdade de Medicina de São José do Rio Preto (FAMERP), Avenida Brigadeiro Faria Lima, 5544, São José do Rio Preto, 15090-000 São Paulo Brazil; 2 Universidade Federal da Paraíba (UFPB), João Pessoa, Paraíba Brazil; 3 Centro Universitário Uninorte (UNINORTE), Rio Branco, Acre Brazil; 4 Universidade Federal de Alagoas (UFAL), Arapiraca, Alagoas Brazil; 5 Universidade Federal de Pernambuco (UFPE), Recife, Pernambuco Brazil; 6 Far Eastern Federal University (FEFU), Vladivostok, Primorsky Krai Russia; 7 Universidade do Estado da Bahia (UNEB), Salvador, Bahia Brazil; 8 Escola Bahiana de Medicina e Saúde Pública (EBMSP), Salvador, Bahia Brazil; 9 Universidade Federal de Santa Catarina (UFSC), Florianópolis, Santa Catarina Brazil

**Keywords:** Chronic kidney disease, Cognitive impairment, Glymphatic system, Choroid plexus, Alzheimer disease biomarkers, Blood–brain barrier

## Abstract

**Background:**

Cognitive impairment is common in chronic kidney disease (CKD), yet vascular, uremic, dialysis-related, and neurodegenerative mechanisms are often considered separately. We propose an integrated kidney–brain tissue-environment framework to support neurological interpretation of cognitive, imaging, and biomarker findings in CKD.

**Methods:**

We conducted a narrative synthesis of clinical, neuroimaging, experimental, and biomarker studies retrieved from PubMed/MEDLINE, Embase, Scopus, and Web of Science through June 2026, focusing on barrier dysfunction, choroid plexus remodeling, cerebrospinal fluid dynamics, neurovascular coupling, white-matter injury, glymphatic-related imaging markers, and Alzheimer disease-related plasma biomarkers.

**Results:**

CKD is associated with convergent vascular, inflammatory, toxic, barrier, and neurofluid-related abnormalities that may contribute to a mixed cognitive phenotype. Human studies report choroid plexus enlargement, lower diffusion tensor image analysis along the perivascular space (DTI-ALPS) indices, altered cerebrospinal fluid-related signal coupling, and neurovascular decoupling; however, evidence is predominantly cross-sectional and does not establish temporal or causal sequencing. Choroid plexus enlargement is morphologically nonspecific, while DTI-ALPS reflects a composite tissue environment rather than direct glymphatic flow. Reduced kidney function may also increase plasma phosphorylated tau, amyloid-β, neurofilament light chain, and glial fibrillary acidic protein concentrations. Ratio-based measures may attenuate, but do not uniformly eliminate, kidney-related confounding.

**Conclusions:**

CKD should be regarded as an active modifier of cognitive phenotype, neuroimaging interpretation, and plasma biomarker assessment. The proposed framework is testable rather than causal and supports reporting kidney function, dialysis context, and multimodal markers when evaluating cognitive decline.

## Introduction: why CKD matters to clinical neurology

Cognitive impairment is common across the stages of chronic kidney disease (CKD) and becomes particularly relevant in kidney failure, affecting memory, executive function, attention, functional independence, treatment adherence, and quality of life [1–3]. For neurologists, CKD should be treated as an active modifier of cognitive phenotype, magnetic resonance imaging (MRI) interpretation, dialysis-related brain vulnerability, and plasma biomarker accuracy rather than as a passive comorbidity. Contemporary models of the kidney–brain interface have moved the discussion beyond the nonspecific concept of “uremia,” incorporating uremic toxin retention, endothelial and blood–brain barrier (BBB) injury, neuroinflammation, white-matter damage, and the possible acceleration of neurodegenerative pathways [1, 2, 4, 5]. Accordingly, CKD-associated cognitive dysfunction may overlap with, amplify, or confound familiar neurological syndromes, including vascular cognitive impairment and biomarker trajectories resembling those observed in Alzheimer disease (AD) [1, 2, 4].

Most reviews still organize CKD-associated cognitive dysfunction around three explanatory axes: vascular disease, uremic neurotoxicity, and dialysis-related factors [1–5]. This taxonomy remains useful, but it fragments mechanisms that are likely to converge biologically. Uremic solutes may alter BBB transport and inflammatory signaling [4, 6–8]; microvascular disease may impair neurovascular coupling and perivascular flow [1, 2, 9]; and dialysis may modify, without necessarily normalizing, central nervous system (CNS) homeostasis [2, 10, 11]. What remains insufficiently integrated is the possibility that CKD may be associated with convergent alterations in systemic solute handling, BBB and blood–cerebrospinal fluid (CSF) barrier biology, choroid plexus (CP) structure, CSF dynamics, glymphatic-related imaging markers, white-matter integrity, and cognitive function. This rationale is supported by prior CKD cognitive reviews and emerging CP/glymphatic evidence [2, 4, 11–15]. The kidney–brain clearance framework proposed here does not replace vascular or toxic models; rather, it reorganizes them as interacting inputs into a barrier, neurovascular, and brain-clearance phenotype.

This review therefore proposes a kidney–brain clearance framework for CKD-associated cognitive decline, focusing on CP remodeling, CSF dynamics, glymphatic-related imaging markers, neurovascular coupling, and blood-based biomarkers of neurodegeneration. We first reconsider uremic neurotoxicity as a disorder of barrier integrity and neural networks; we then examine the CP as a blood–CSF interface that may link systemic kidney failure to altered CSF composition and neurofluid homeostasis. Finally, we synthesize human neuroimaging evidence on glymphatic-related and neurofluid imaging alterations and discuss how CKD may complicate the interpretation of plasma biomarkers. The aim is not to assert a single causal pathway, but to delineate a testable neurological framework connecting kidney failure, brain-clearance physiology, neuroimaging phenotypes, and cognitive vulnerability. Unlike previous reviews that have primarily emphasized vascular injury, uremic neurotoxicity, dialysis-related factors, or broad kidney–brain mechanisms, this review specifically integrates brain-clearance physiology, choroid plexus remodeling, glymphatic and neurovascular imaging markers, Alzheimer disease biomarker confounding, and a practical neurologic checklist into a single clinically oriented framework.

**Fig. 1:**
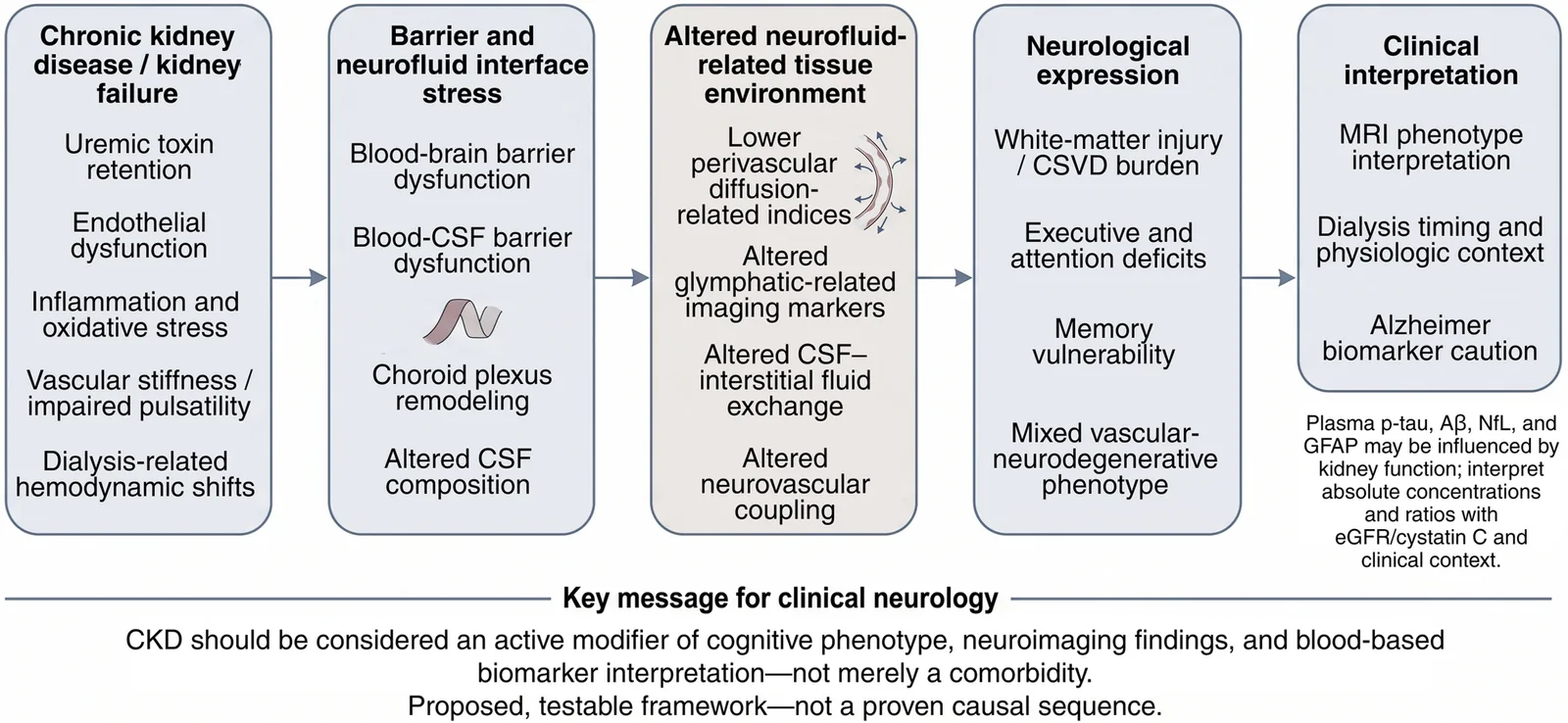
Proposed kidney–brain tissue-environment framework in chronic kidney disease. Chronic kidney disease and kidney failure may modify cognitive vulnerability through convergent systemic, vascular, barrier, neurofluid-related, white-matter, and biomarker-associated mechanisms. Uremic toxin retention, endothelial dysfunction, inflammation, oxidative stress, vascular stiffness, altered pulsatility, and dialysis-related hemodynamic or fluid shifts may coexist with blood–brain barrier dysfunction, blood–CSF interface stress, choroid plexus remodeling, and altered CSF composition. These processes may be associated with lower perivascular diffusion-related indices, altered glymphatic-related imaging markers, altered CSF–interstitial fluid exchange, and neurovascular dysregulation. The neurological phenotype may include white-matter injury, cerebral small-vessel disease, executive and attention deficits, memory vulnerability, and mixed vascular–neurodegenerative presentations. Plasma p-tau, amyloid-β, NfL, and GFAP concentrations may be influenced by kidney function and should be interpreted with eGFR, cystatin C, ratio-based measures where appropriate, and the broader clinical context. DTI-ALPS is a structure-dependent, ratio-based marker of localized directional diffusivity and not a direct measurement of glymphatic flow. Arrows indicate hypothesized, non-exclusive associations and should not be interpreted as temporal ordering, causal mediation, or a diagnostic algorithm. **Abbreviations:** Aβ, amyloid-β; CKD, chronic kidney disease; CSF, cerebrospinal fluid; CSVD, cerebral small vessel disease; DTI-ALPS, diffusion tensor imaging analysis along the perivascular space; eGFR, estimated glomerular filtration rate; GFAP, glial fibrillary acidic protein; MRI, magnetic resonance imaging; NfL, neurofilament light chain; p-tau, phosphorylated tau

Throughout this review, “brain clearance” is used as a broad conceptual term rather than as a synonym for glymphatic flow. The proposed framework encompasses the brain tissue environment generated by barrier function, vascular pulsatility, CSF–interstitial fluid exchange, localized perivascular water mobility, white-matter microstructure, and systemic metabolic clearance. Glymphatic-related imaging markers therefore represent one component of this framework and should not be interpreted as direct measurements of a single whole-brain waste-clearance pathway [16, 17]. The hypothesized sequence linking CKD, choroid plexus remodeling, altered neurofluid physiology, and cognitive decline is consequently presented as a testable conceptual pathway rather than as an established temporal or causal cascade. This proposed kidney–brain clearance framework is summarized in Fig. 1.

## Methods of the narrative synthesis

We conducted a narrative review of the literature to synthesize mechanistic, neuroimaging, clinical, and biomarker evidence linking chronic kidney disease to cognitive decline. PubMed/MEDLINE, Embase, Scopus, and Web of Science were searched up to June 2026 using combinations of terms related to chronic kidney disease, kidney failure, dialysis, cognitive impairment, dementia, Alzheimer disease, choroid plexus, cerebrospinal fluid, glymphatic system, blood–brain barrier, blood–cerebrospinal fluid barrier, neurovascular coupling, white-matter hyperintensities, perivascular spaces, diffusion tensor imaging along the perivascular space, resting-state functional MRI, arterial spin labeling, amyloid-β, phosphorylated tau, and neurofilament light chain. We prioritized human neuroimaging studies, clinical biomarker studies, mechanistic experimental work with direct relevance to kidney–brain biology, and recent reviews or consensus documents in cognitive neurology. Because the objective was conceptual integration rather than pooled effect estimation, no meta-analysis was performed. This was a narrative, non-systematic synthesis; therefore, study selection was interpretive, no formal risk-of-bias assessment was performed, and the conclusions should be read as a clinically oriented framework rather than as a graded evidence statement.

## CKD-associated cognitive decline: beyond vascular and uremic models

CKD is associated with an increased risk of cognitive impairment, and the classic model invoked to explain this relationship brings together uremic solute retention, inflammation, oxidative stress, acid–base and electrolyte disturbances, anemia, endothelial dysfunction, and microvascular injury as convergent axes of neurotoxicity [1–5]. Reframed in neurological terms, these factors affect defined cellular targets. At the BBB, indoxyl sulfate (IS) activates the aryl hydrocarbon receptor (AhR) in cerebral endothelium and promotes BBB disruption with cognitive impairment in CKD models, an effect abolished in AhR-/- mice [6]; in humans with end-stage renal disease (ESRD), BBB permeability correlated inversely with Montreal Cognitive Assessment (MoCA) scores [18]. At the level of neuronal excitability, uremic exposure induces microglial potassium efflux through the calcium-activated potassium channel KCa3.1 and reduces neuronal potassium turnover [7]. In microglial activation, IS increases reactive oxygen species, reduces the nuclear factor erythroid 2-related factor 2 (Nrf2)-mediated antioxidant response, and upregulates inducible nitric oxide synthase (iNOS), cyclooxygenase-2 (COX-2), tumor necrosis factor-α (TNF-α), and interleukin-6 (IL-6), culminating in neuronal death [8], while potassium efflux primes interleukin-1β (IL-1β) maturation, which injures neurons through interleukin-1 receptor type 1 (IL-1R1) [7]. Finally, at the level of synaptic function and network integrity, IS accumulates in the brainstem, reduces regional monoamines, and produces apathy and spatial memory impairment [19].

Although the uremic neuroinflammation model explains part of the cognitive dysfunction observed in CKD, it does not satisfactorily organize recent neuroimaging and biomarker findings. Studies using diffusion tensor image analysis along the perivascular space (DTI-ALPS) have shown reduced indices in patients with CKD, including those at early stages and even in the absence of neurological symptoms [11, 13–15]. These findings indicate an alteration in localized periventricular directional diffusivity, but they should not be equated with directly measured glymphatic impairment because the ALPS index is a ratio-based and structure-dependent diffusion marker influenced by white-matter architecture, extracellular water, vascular geometry, acquisition conditions, and its individual numerator and denominator components [16, 17]. In the same direction, patients with ESRD exhibit not only reduced DTI-ALPS indices but also increased CP volume [12]—the structure that governs the blood–CSF barrier [20, 21]—an observation hypothesized to reflect choroid plexus remodeling in the setting of an altered neurofluid-related tissue environment; compensatory remodeling is only one of several possible and currently unproven interpretations. The DTI-ALPS index has also been associated with dementia severity in patients with AD [22] and with cognitive performance in CKD [14]. In parallel, multimodal approaches using resting-state functional MRI (rs-fMRI), quantitative susceptibility mapping (QSM), and arterial spin labeling (ASL) reveal neurovascular uncoupling linked to cognitive decline in these patients [9]. Added to this is evidence that reduced kidney function increases plasma concentrations of neurodegenerative biomarkers, affecting their diagnostic accuracy [23–28]. This is the necessary conceptual bridge: impaired peripheral solute clearance and altered central brain-clearance physiology may converge in CKD, but this relationship should not be interpreted as a direct causal chain. Shared upstream drivers, including aging, hypertension, diabetes, endothelial dysfunction, inflammation, and vascular injury, may simultaneously affect kidney function, cerebral small-vessel disease, barrier integrity, perivascular transport, and cognition [1–3].

We therefore propose reframing cognitive impairment in CKD through a kidney–brain clearance framework, understood as the convergence of interdependent vascular, uremic, barrier, neurovascular, and brain-clearance mechanisms that, in isolation, escape the uremic neuroinflammation model. At the systemic level, deficient solute clearance increases the burden of uremic toxins [4, 5] and may influence circulating concentrations of neurodegeneration-related proteins, including amyloid-β species, whose plasma levels are associated with kidney function through mechanisms that may include altered peripheral elimination and CKD-related systemic or cerebral injury [23–28]. At the interfaces, dysfunction of the BBB and blood–CSF barrier—the latter governed by the CP—alters solute selectivity and transit and modulates CSF production and composition [6, 18, 20, 21], an axis already altered in ESRD, in which greater CP volume coexists with a lower DTI-ALPS index [12]. Experimental models support the possibility that reduced arterial pulsatility and disruption of aquaporin-4 (AQP4) organization may alter periarterial CSF influx and interstitial solute movement [29, 30]. Chronic cerebral hypoperfusion can also shift amyloid-β oligomer equilibria toward more aggregation-prone species [31]. However, the magnitude, direction, and physiological relevance of bulk or convective transport in the human brain remain debated, and these findings do not establish that CKD causes cerebral amyloid retention through a glymphatic mechanism [16, 17, 30]. A continuum therefore emerges in which altered glymphatic-related physiology may represent one component of a broader brain tissue and neurofluid framework.

## Barrier dysfunction and the choroid plexus as a kidney–brain interface

The CP is not merely a producer of CSF. Its epithelium, joined by tight junctions over fenestrated capillaries, constitutes the blood–CSF barrier and functions as a secretory, metabolic, and immunological interface, regulating solute transport, CSF composition, and immune surveillance [20, 21]. This position enables the CP to influence CSF composition and CSF–interstitial fluid exchange [20, 21]. Experimental models indicate that CP dysfunction can alter amyloid-β handling [32]; however, direct evidence that human CKD causes cerebral amyloid retention through CP dysfunction remains unavailable. In aging, multiparametric MRI reveals CP enlargement, hypoperfusion, and increased diffusivity, compatible with vascular and microstructural remodeling [33]. In AD, greater CP volume and abnormal permeability are associated with worse memory, executive function, and global cognition, while epithelial alterations, fibrosis, and amyloid or tangle-like deposits support its connection with neurodegeneration [20, 34]. CP enlargement has also been associated with WMH progression, with the DTI-ALPS index identified as a statistical mediator in the reported analysis [35].

Choroid plexus enlargement is morphologically nonspecific and cannot, from volumetry alone, be assigned to a single biological process [33, 34, 36]. Potential contributors include compensatory or degenerative epithelial hypertrophy, mitochondrial or oncocytic remodeling, stromal and basement-membrane expansion, fibrosis, altered vascular density or permeability, interstitial fluid accumulation, ventricular geometry, and combinations of these processes [33, 34, 36]. Human aging studies have demonstrated larger CP volume alongside lower perfusion and altered diffusion characteristics, whereas histopathological studies support epithelial degeneration, compensatory hypertrophy of residual cells, mitochondrial remodeling, and stromal fibrosis [33, 36]. Accordingly, CP enlargement should not be labeled as secretory hypertrophy, edema, compensatory remodeling, or direct uremic injury without convergent functional, permeability, diffusion, histological, or longitudinal evidence.

The most relevant human imaging evidence in ESRD comes from Park et al.: 40 patients with ESRD—20 receiving hemodialysis (HD) and 20 receiving peritoneal dialysis (PD)—had greater normalized CP volume than 42 healthy controls (1.392% versus 1.138%) and a lower DTI-ALPS index, an indirect marker of perivascular transport [12]. The co-occurrence of greater CP volume and a lower DTI-ALPS index supports a candidate multimodal kidney–brain tissue-environment phenotype, but does not establish that CP enlargement caused altered CSF dynamics or reduced perivascular transport. Clinically, 72.5% of patients had cognitive impairment, and CP volume correlated inversely with word-list recognition, linking it specifically to verbal memory [12]. Interpretation requires caution: in this cross-sectional analysis, CP volume did not distinguish patients with and without cognitive impairment and was not associated with executive tests [12]. The executive dimension is supported by parallel evidence, as studies in ESRD and across CKD have shown reduced DTI-ALPS indices, more pronounced among cognitively impaired individuals [14, 15]. Thus, CP volumetry should be interpreted alongside diffusion-derived, CSF-related, and neurovascular research markers, none of which independently measures brain clearance.

Clinical neurology has traditionally emphasized hippocampal or cortical atrophy, WMH, and microbleeds; CP volume adds a candidate imaging phenotype that may reflect aspects of systemic toxicity, barrier stress, vascular remodeling, and altered neurofluid homeostasis. In CKD, CP enlargement may represent a nonspecific imaging marker of blood–CSF interface remodeling. Its biological substrate and functional consequences remain uncertain and may involve aging, systemic vascular burden, inflammation, epithelial alteration, fibrosis, permeability changes, or altered CSF dynamics rather than a CKD-specific clearance lesion [12, 33, 34, 36]. This hypothesis is plausible because IS is associated with worse executive performance in early CKD and, experimentally, disrupts the BBB through AhR activation; p-cresyl sulfate induces oxidative stress, neuroinflammation, and neurodegeneration in uremic animals [6, 37, 38]. However, it has not yet been demonstrated that these solutes directly cause human CP hypertrophy. In parallel, loss of meningeal lymphatic drainage reduces memory and intensifies amyloid retention, providing a biological basis for reduced cognitive resilience when efflux systems fail [39]. The CP therefore emerges as a bridge biomarker to be validated through longitudinal MRI, DTI-ALPS, and plasma/CSF metabolomics.

## Glymphatic-related and neurofluid imaging markers in CKD: human evidence

The first relevant signal of altered glymphatic-related directional diffusivity in CKD emerged from neurologically asymptomatic patients at early stages of disease [13]. In a pioneering prospective study, Heo et al. compared the DTI-ALPS index between 18 patients with early CKD (stages 3a to 4, with no history of neurological disease) and 18 healthy controls matched by age and sex, demonstrating a statistically significant reduction in the index among patients with CKD compared with controls (1.259 ± 0.199 vs. 1.477 ± 0.232; *p* = 0.004) [13]. The lower DTI-ALPS index was observed before clinically evident cognitive impairment, raising the hypothesis that alterations in the perivascular tissue environment may precede overt neurological manifestations [13]. Nevertheless, the cross-sectional comparison does not demonstrate individual temporal progression or establish that a clearance abnormality preceded cognitive decline [13, 16, 17]. Although correlation analyses did not identify associations between the DTI-ALPS index and laboratory parameters such as hemoglobin, estimated glomerular filtration rate (eGFR), or creatinine, the mechanistic hypotheses proposed by the authors include mechanisms previously described in the literature, such as arterial stiffness and reduced cerebral blood flow induced by vascular injury—both capable of compromising arterial pulsation-mediated glymphatic influx—as well as possible reduction in AQP4 expression, a transmembrane channel fundamental to CSF dynamics in the perivascular space [13, 29, 30]. These data therefore provided early human evidence of altered localized periventricular directional diffusivity in CKD, without directly demonstrating glymphatic flow, whole-brain clearance, or temporal causality [13, 16, 17]. The main human neuroimaging studies supporting this kidney–brain clearance framework are summarized in Table 1.

**Table 1:** Human neuroimaging evidence linking CKD to brain-clearance and neurofluid phenotypes

Study	Population/design	Imaging target	Key finding	Clinical/cognitive link	Interpretation and limitations
Heo et al. [13]	Early CKD; prospective; *n* = 18 CKD stages 3a-4 and *n* = 18 controls; neurologically asymptomatic.	DTI-ALPS index.	Lower DTI-ALPS in CKD: 1.259 ± 0.199 vs. 1.477 ± 0.232; *p* = 0.004.	Signal detected before overt neurologic symptoms; no clear correlation with eGFR, creatinine, or hemoglobin.	Supports early perivascular diffusion abnormality, but not causality or a validated screening threshold.
Heo et al. [15]	ESRD on dialysis; prospective; *n* = 49 ESRD and *n* = 38 controls; HD and PD included.	DTI-ALPS index.	Lower DTI-ALPS in ESRD: 1.460 vs. 1.632; *p* = 0.003.	DTI-ALPS correlated with PTH (*r* = 0.357; *p* = 0.011), not with dialysis vintage.	Pioneering ESRD evidence; modality-specific inference limited by sample size and cross-sectional design.
Lee et al. [11]	ESKD assessed before and 3 months after RRT initiation.	Serial DTI-ALPS index around dialysis initiation.	Predialysis ESKD had lower DTI-ALPS than controls; no robust improvement after RRT initiation: 1.342 vs. 1.262; *p* = 0.386.	Persistence despite improved conventional uremic markers suggests incomplete neurologic normalization.	Suggests that conventional RRT initiation may not rapidly normalize perivascular diffusion; timing and modality effects remain unresolved.
Xiong et al. [10]	ESKD before and after a single HD session; prospective; *n* = 25 ESKD and *n* = 27 controls.	Pre-/post-HD DTI-ALPS index.	ESKD had lower ALPS index than controls: 1.379 ± 0.154 vs. 1.469 ± 0.090; *p* = 0.023; no acute HD change: 1.379 vs. 1.387; *p* = 0.493.	ALPS index correlated with prealbumin (*r* = 0.663; FDR-corrected *p* = 0.036).	Supports the need to standardize MRI timing, because a single HD session may not acutely normalize perivascular diffusion.
Park et al. [12]	ESRD; *n* = 40 patients (20 HD, 20 PD) and *n* = 42 controls.	Normalized CP volume plus DTI-ALPS index.	ESRD showed larger normalized CP volume (1.392% vs. 1.138%) and lower DTI-ALPS index.	Cognitive impairment in 72.5%; CP volume inversely related to word-list recognition.	Provides human imaging evidence of a CPV–CKD association; CPV should be interpreted alongside diffusion-derived and CSF-related measures, none of which independently establishes brain clearance.
Ko et al. [14]	CKD across stages; prospective; *n* = 56 CKD and *n* = 38 controls; CKD subgrouped by cognition.	DTI-ALPS plus graph-theory structural connectivity.	Lower DTI-ALPS in CKD: 1.436 vs. 1.632; *p* < 0.001; lower in CKD with cognitive impairment: 1.338 vs. 1.494; *p* = 0.031.	DTI-ALPS, not structural connectivity alone, separated cognitive subgroups.	Associates a diffusion-derived imaging marker with cognitive status while preserving vascular, microstructural, and network-level confounding.
Yu et al. [40]	Nondialysis-dependent ESRD, including diabetic kidney disease phenotype.	DTI-ALPS and cognitive/clinical correlation.	The DKD phenotype showed lower DTI-ALPS indices than non-DKD ESRD in the reported analyses.	DTI-ALPS metrics were associated with clinical characteristics and cognitive performance.	Supports imaging-phenotype heterogeneity; diabetes and vascular-metabolic burden are potential contributors but causal attribution is not possible.
Du et al. [41]	CKD; *n* = 61 CKD (22 non-dialysis-dependent, 39 dialysis-dependent) and *n* = 43 controls.	DTI-ALPS, CPV, and white-matter network topology.	CKD had reduced DTI-ALPS (*p* = 0.001) and increased CPV (*p* < 0.001), with no major dialysis-subgroup difference.	Cross-sectional mediation analyses suggested that white-matter small-worldness may partly link DTI-ALPS/CPV metrics to depression/anxiety scores.	Shows cross-sectional multimodal co-occurrence of CP enlargement, perivascular diffusion abnormalities, and network-level measures without establishing temporal or causal relationships.
Song et al. [42]	CKD and controls; *n* = 26 CKD and *n* = 16 controls.	DTI with prolonged echo time and low b-value to estimate periarterial CSF influx.	Reduced periarterial subarachnoid FA in CKD (*p* = 0.008).	FA correlated positively with MoCA (*r* = 0.846; *p* < 0.001); axial diffusivity correlated negatively with cognition.	Conference-abstract evidence from a small cohort; the unusually strong FA–MoCA correlation may be unstable and should not be used for causal, diagnostic, or prognostic inference without full peer-reviewed publication and independent replication.
Yang et al. [43]	CKD with and without cognitive impairment; *n* = 29 CKD and *n* = 22 controls.	rs-fMRI gBOLD-CSF coupling.	Reduced gBOLD-CSF coupling in CKD (β=-0.178; *p* = 0.003), with greater reduction in cognitive impairment (β=-0.135; *p* = 0.040).	Links impaired blood-volume-driven CSF oscillations to cognitive phenotype.	Important because it examines CSF-related signal coupling complementary to diffusion-derived proxies, without directly measuring solute clearance or glymphatic flow.
Luo et al. [44]	ESRD with mild cognitive impairment; *n* = 68 ESRD-MCI and *n* = 65 controls.	CPV, PVS volume fraction, DTI-ALPS, and NVC metrics.	CSF-circulation abnormalities coexisted with global/regional NVC impairment.	Mediation analyses suggested NVC may connect CSF-circulation dysfunction to cognitive decline.	Supports an integrated neurofluid-NVC model; cross-sectional mediation is hypothesis-generating, not causal proof.
Yang et al. [45]	Dialysis patients; HD and PD compared with controls.	ALFF-CBF coupling plus DTI-ALPS index.	HD and PD showed lower NVC metrics and lower DTI-ALPS than controls; no clear HD-PD difference.	Positive association between ALFF-CBF coupling and DTI-ALPS within dialysis groups.	Makes dialysis status a physiologic modifier of imaging interpretation, not just a comorbidity label.

Abbreviations: *AD* Alzheimer disease, *ALFF-CBF* amplitude of low-frequency fluctuation-cerebral blood flow coupling, *CKD* chronic kidney disease, *CP* choroid plexus, *CPV* choroid plexus volume, *CSF* cerebrospinal fluid, *DKD* diabetic kidney disease, *DTI-ALPS* diffusion tensor imaging analysis along the perivascular space, *ESKD/ESRD* end-stage kidney disease/end-stage renal disease, *FA* fractional anisotropy, *FDR* false discovery rate, *gBOLD* global blood oxygen level-dependent signal, *HD* hemodialysis, *MoCA* Montreal Cognitive Assessment, *NVC* neurovascular coupling, *PD* peritoneal dialysis, *PTH* parathyroid hormone, *PVS* perivascular spaces, *RRT* renal replacement therapy, *rs-fMRI* resting-state functional MRI

Note: DTI-ALPS is a ratio-based, spatially fixed-point marker of localized directional diffusivity rather than a direct measurement of glymphatic flow. A lower index may result from a reduction in numerator diffusivity, an increase in denominator diffusivity, or both, and may reflect white-matter microstructure, extracellular free water, vascular geometry, head position, acquisition and processing factors, dialysis-related fluid shifts, and neurofluid-related tissue changes. DTI-ALPS findings should therefore be interpreted as nonspecific components of the brain tissue environment and not as proof of whole-brain clearance failure or causality [10, 16, 17]

A major interpretive caveat is that DTI-ALPS is neither a tracer-based measurement nor a direct quantification of glymphatic flow [16, 17]. It evaluates directional diffusivity within anatomically defined periventricular white-matter regions [16, 17]. Its numerator incorporates x-axis diffusivity in projection- and association-fiber regions, whereas its denominator incorporates orthogonal diffusivity components in those regions [16, 17]. Consequently, the same reduction in the ALPS ratio may result from a lower numerator, a higher denominator, or simultaneous changes in both, each potentially representing a different tissue process [16, 17].

The index is additionally influenced by white-matter microstructure, crossing fibers, fiber orientation, extracellular geometry and free water, vascular anatomy, head position, region-of-interest placement, acquisition parameters, preprocessing procedures, and age-related diffusion changes [16, 17]. Taoka et al. therefore proposed interpreting DTI-ALPS as a structure-dependent “spatially fixed-point diffusion biomarker” reflecting a composite brain tissue environment rather than as a direct measurement of whole-brain glymphatic clearance [17]. In CKD and kidney failure, volume overload, edema, osmolality changes, and dialysis-related extracellular fluid shifts may further alter numerator or denominator diffusivity independently of neurofluid transport [10].

At the more advanced end of the kidney disease spectrum, Lee et al. prospectively investigated longitudinal changes in the DTI-ALPS index in patients with ESRD before and three months after initiation of renal replacement therapy (RRT) [11]. The results showed a significantly lower DTI-ALPS index in predialysis ESRD than in healthy controls (DTI-ALPS index: 1.342 vs. 1.633; *p* = 0.003) [11]. Crucially, the DTI-ALPS index did not differ significantly between the pre- and postdialysis-initiation periods (1.342 vs. 1.262; *p* = 0.386), indicating that initiation of conventional RRT—with reduction of low-molecular-weight uremic toxins and improvement in parameters such as serum urea and bicarbonate—did not produce a statistically significant change in the DTI-ALPS index [11]. The absence of a significant short-term change after dialysis initiation suggests that the ALPS signal is not determined solely by conventional small-solute clearance; persistent tissue alterations, limited statistical power, treatment timing, and fluid-related effects remain alternative explanations. The authors hypothesized that poorly dialyzable protein-bound uremic toxins may contribute to persistent barrier or perivascular abnormalities; this mechanism has not been directly demonstrated by the imaging data [11]. This finding reinforces the hypothesis that brain injury associated with advanced CKD is not simply uremic in the classic sense, but involves mechanisms independent of dialysis efficiency.

Subsequent studies have identified cross-sectional associations among the DTI-ALPS index, white-matter network measures, and cognitive performance [14, 41]. In a prospective study published in 2024, Ko et al. evaluated 56 patients with CKD and 38 controls, demonstrating that the DTI-ALPS index was significantly lower in patients with CKD (1.436 vs. 1.632; *p* < 0.001) and that those with cognitive impairment had even lower indices than those without impairment (1.338 vs. 1.494; *p* = 0.031), whereas structural connectivity metrics did not differ between cognitive subgroups—showing that the DTI-ALPS index, but not the assessed structural-connectivity metrics, differentiated the cognitive subgroups in that cohort [14]. This finding does not establish physiological specificity or identify DTI-ALPS as a determinant of cognition [14, 16, 17]. Expanding this framework, Du et al. integrated the DTI-ALPS index with measurement of choroid plexus volume (CPV) and graph-theory analysis of white-matter networks in a multiparametric study published in 2025 in Quantitative Imaging in Medicine and Surgery, including 61 patients with CKD (22 non–dialysis-dependent and 39 dialysis-dependent) and 43 controls [41]. Patients with CKD had reduced DTI-ALPS indices (*p* = 0.001) and increased CPV (*p* < 0.001), with no significant differences between dialysis-dependent and non–dialysis-dependent subgroups in either the DTI-ALPS index or CPV, a result consistent with the findings of Lee et al. and Ko et al. [11, 14, 41]. Cross-sectional mediation analyses suggested that white-matter network small-worldness (σ) may partly link DTI-ALPS and CPV metrics to depression and anxiety scores; however, these analyses should be interpreted as hypothesis-generating and do not establish temporal ordering or causal mediation between DTI-ALPS/CPV measures, network topology, and neuropsychiatric outcomes in CKD [41].

The field is progressively moving toward multimodal, but still indirect, in vivo characterization of neurofluid-related and brain tissue physiology, with the emergence of techniques complementary to DTI-ALPS. In a study presented at the International Society for Magnetic Resonance in Medicine (ISMRM) 2024 meeting, Song et al. developed a novel approach to measure CSF influx into the subarachnoid space along the middle cerebral artery using low b-value diffusion tensor imaging with prolonged echo time (DTI_low-b), applied to 26 patients with CKD and 16 controls [42]. Patients with CKD were reported to have reduced fractional anisotropy (FA) in the periarterial subarachnoid space (*p* = 0.008), with a positive correlation between FA and MoCA scores (*r* = 0.846; *p* < 0.001) and a negative correlation between axial diffusivity and cognition (*r* = − 0.395; *p* = 0.045) [42]. Because this finding derives from a conference abstract involving only 26 patients with CKD and 16 controls, the reported FA–MoCA correlation (*r* = 0.846) is potentially imprecise and vulnerable to selection effects, multiplicity, and effect-size inflation. It should therefore be regarded as preliminary and hypothesis-generating until the full methods, confidence intervals, correction procedures, and independent peer-reviewed replication are available.

In parallel, Yang et al. assessed global blood oxygen level-dependent–cerebrospinal fluid (gBOLD–CSF) coupling in the fourth ventricle using rs-fMRI in 29 patients with CKD (19 with and 10 without cognitive impairment) and 22 controls, demonstrating that gBOLD-CSF coupling was significantly reduced in patients with CKD (β = -0.178; *p* = 0.003) and even more impaired in those with cognitive impairment (β = -0.135; *p* = 0.040) [43]. This measure quantifies the temporal relationship between global blood oxygen level-dependent signal fluctuations and the CSF signal in the fourth ventricle [43]. It is thought to sample blood-volume–CSF oscillatory relationships, but it does not directly measure solute clearance or glymphatic flow [43]. Lower coupling in CKD may reflect interacting vascular, respiratory, sleep-state, hemodynamic, and neurofluid factors; attribution to endothelial dysfunction, altered AQP4 organization, or uremic toxins remains mechanistic inference rather than a finding directly demonstrated by the imaging measurement [43].

Together, these emerging techniques—DTI_low-b, gBOLD-CSF coupling, and DTI-ALPS—are not yet part of clinical practice, but they provide research-level and complementary measurements of localized tissue diffusion, CSF-related signal coupling, and vascular physiology [16, 17, 42, 43]. None should currently be regarded as a stand-alone measurement of glymphatic clearance or as a diagnostic test for an individual patient [16, 17, 43]. These modalities remain research tools and should not be interpreted as diagnostic tests for individual patients until acquisition protocols, reproducibility, CKD-specific thresholds, and clinically meaningful longitudinal changes are validated.

## Neurovascular coupling, white-matter injury, and cognitive phenotypes

Increasing evidence indicates that the kidney and brain are linked through homologous low-resistance microvascular systems, rendering both organs vulnerable to endothelial dysfunction, arterial stiffness, oxidative stress, and chronic inflammation [1, 2]. This shared susceptibility has led to the concept of a brain–kidney axis, whereby CKD contributes to the development of cerebral small vessel disease (CSVD) [1, 2]. In a large multicenter study, higher CKD risk grades were associated with greater CSVD burden, and WMH, lacunar infarctions, and cerebral microbleeds were significantly more frequent among patients with CKD [46]. Cognitive impairment is highly prevalent in CKD and is characterized predominantly by deficits in executive function, attention, and processing speed [3]. Importantly, this association should not be interpreted as CKD-specific causality. Aging, hypertension, diabetes, and systemic vascular risk may simultaneously drive kidney dysfunction, cerebral small-vessel disease, blood–brain barrier injury, perivascular-space abnormalities, and cognitive decline [1, 2, 46]. The clinical challenge is therefore not to assign a single cause, but to recognize CKD as a modifier within a mixed vascular, metabolic, and brain-clearance phenotype.

Beyond structural white-matter injury, emerging evidence suggests that impaired neurovascular regulation may contribute to cognitive dysfunction in CKD [9, 44, 45]. Glymphatic-related physiology is thought to involve interactions among CSF circulation, vascular pulsatility, AQP4-mediated fluid transport, and neurovascular coupling (NVC); however, the contribution of each component to human brain solute clearance remains incompletely resolved [29, 30, 44, 45]. A recent study in patients with ESRD and mild cognitive impairment demonstrated abnormalities in CSF circulation, including increased CPV and perivascular space volume fraction, together with reduced DTI-ALPS indices, indicating a coexisting abnormality in a nonspecific diffusion-derived neurofluid marker [44]. These alterations were accompanied by reduced global and regional NVC metrics, and cross-sectional mediation analyses identified NVC metrics as a possible statistical indirect pathway between the imaging variables and cognition [44]. Such analyses do not establish temporal ordering, biological mediation, or directionality. Collectively, these findings support the co-occurrence of CSF-related, diffusion-derived, and neurovascular abnormalities within an integrated ESRD imaging phenotype [44]. Longitudinal studies are required to determine whether one process precedes, mediates, or causes another.

Recent evidence further supports an association between NVC metrics and the DTI-ALPS index in patients with ESRD receiving dialysis [45]. Yang et al. demonstrated that both HD and PD patients exhibited reduced amplitude of low-frequency fluctuation–cerebral blood flow (ALFF-CBF) coupling coefficients and lower DTI-ALPS indices compared with healthy controls, showing concurrent abnormalities in neurovascular coupling and the DTI-ALPS index [45]. No significant differences were observed between dialysis modalities [45]. This negative comparison does not establish the uremic milieu as the dominant mechanism because modality-specific differences may have been obscured by sample size, residual confounding, dialysis vintage, treatment intensity, or patient selection. Significant positive correlations between ALFF-CBF coupling coefficients and DTI-ALPS indices were identified in both dialysis groups, supporting an association between neurovascular decoupling and a lower DTI-ALPS index [17, 45]. For neurologists, this suggests that dialysis status should be recorded not only as a medical comorbidity but also as a physiological modifier of neurovascular and glymphatic imaging markers.

## Alzheimer disease biomarkers in CKD: a diagnostic interpretation problem

Blood-based biomarkers for Alzheimer disease and neurodegeneration are moving from research into clinical neurology, but CKD introduces a major interpretive vulnerability: reduced kidney function may increase circulating concentrations of amyloid-β, phosphorylated tau, neurofilament light chain (NfL), and glial fibrillary acidic protein (GFAP) independently of central Alzheimer pathology or primary neurodegeneration [23–28]. In this setting, the peripheral compartment does not function as a neutral transport medium, but rather as an environment subject to systemic solute retention [23–28]. A landmark study published in Nature Medicine demonstrated that comorbidities—notably kidney dysfunction—increase plasma levels of phosphorylated tau (p-tau) isoforms, such as p-tau181 and p-tau217 [25]. The authors showed that the magnitude of biomarker elevation associated with kidney dysfunction can rival differences attributed to brain amyloid-β positivity, establishing an important diagnostic confounder for plasma biomarker interpretation [25].

Given this analytical vulnerability, recent investigations have extended these findings by demonstrating the practical impact of eGFR on diagnostic testing [23, 24, 26–28]. Because reduced eGFR is associated with higher plasma concentrations of several neurodegeneration-related proteins, applying uniform reference values may reduce specificity and increase the risk of false-positive classification in patients with CKD [23–28]. Altered peripheral elimination is biologically plausible, but CKD-associated systemic inflammation, vascular brain injury, altered protein metabolism, and true cerebral injury may also contribute, with the relative importance varying across analytes [24–28].

Absolute biomarker concentrations and ratio-based measures, including amyloid-β42/amyloid-β40 (Aβ42/Aβ40) and p-tau217/Aβ42, should be interpreted separately. Because kidney dysfunction may influence both components of a ratio in a partially parallel manner, ratios can attenuate some of the confounding observed with isolated concentrations; however, they should not be assumed to eliminate it uniformly [24]. In the multicenter cohort reported by Mondésert et al., mean ratio-based measures were less strongly influenced by eGFR than individual concentrations, and the p-tau217/Aβ42 ratio maintained relatively stable diagnostic performance across kidney-function groups [24]. Nevertheless, the Aβ42/Aβ40 ratio did not uniformly abolish eGFR-related changes in diagnostic specificity, the eGFR < 60 mL/min/1.73 m² group included only 36 participants, and patients with advanced CKD or kidney failure were underrepresented [24]. Ratios should therefore be considered potentially more robust, rather than kidney-function independent, and require assay-specific validation across CKD stages.

Accordingly, future biomarker studies and clinical algorithms should report contemporaneous eGFR and, when available, cystatin C or combined creatinine–cystatin C eGFR; prespecify kidney-function strata; distinguish absolute concentrations from ratio-based results; assess interactions between eGFR and biomarker performance; and avoid transferring thresholds derived from populations with preserved kidney function to advanced CKD without validation. When plasma results would determine a major diagnosis or disease-modifying treatment, discordant or borderline findings in advanced CKD should be integrated with the clinical phenotype and, when appropriate, confirmed using CSF biomarkers, amyloid or tau positron emission tomography (PET), or a kidney-adjusted algorithm rather than interpreted in isolation.

This intersection between peripheral retention and neurodegenerative diagnosis should not be viewed as a mere laboratory artifact, but as the reflection of a broader clearance vulnerability. In summary, these findings are consistent with an expanded tissue-environment and clearance framework: CKD may simultaneously modify systemic biomarker concentrations, alter BBB and blood–CSF barrier biology, and be associated with abnormalities in neurofluid-related imaging proxies, making the interpretation of peripheral markers and the inference of central pathology substantially more complex. Human imaging studies in CKD and diabetic kidney disease support the coexistence of altered glymphatic-related imaging metrics, altered CSF-related coupling, and cognitive vulnerability [40, 43], but they do not yet prove a unidirectional causal pathway from uremic toxins to CP remodeling or amyloidogenic protein retention. Accordingly, plasma biomarker concentrations in CKD should be interpreted within an integrated framework encompassing kidney-dependent peripheral handling, systemic disease burden, and central nervous system pathology rather than as evidence of a simple peripheral-to-central causal sequence.

Table 3 provides an interpretive taxonomy of the assessed markers according to the biological domain they sample and emphasizes that structural, diffusion-derived, CSF-dynamic, neurovascular, and blood-based measures are complementary but not interchangeable.

## Practical implications for neurologists

In clinical neurology, CKD should prompt three calculated clinical questions when evaluating cognitive complaints. First, is this patient’s cognitive impairment vascular, neurodegenerative, uremic, or mixed in origin? Both vascular and neurodegenerative mechanisms have been proposed to explain CKD-related cognitive decline, and differentiating them at the bedside remains genuinely difficult [1–3]. A growing body of evidence also implicates uremic toxin accumulation, microvascular injury, chronic inflammation, neurovascular dysregulation, and altered brain-clearance physiology as interacting contributors [2, 4, 9, 45], meaning that pure phenotypes are the exception rather than the rule [2]. Second, could CKD itself be altering how the neurologist interprets MRI findings or blood biomarkers? Reduced kidney clearance, fluid shifts, and dialysis-related hemodynamic changes may modify the apparent burden of white-matter disease or biomarker concentrations independently of true neurodegenerative burden [10, 23–28]. Third, is the patient’s cognitive phenotype compatible with a kidney–brain clearance or neurovascular phenotype—that is, do the pattern of deficits and any available imaging suggest altered brain-clearance physiology rather than a purely vascular or degenerative process? A practical checklist for neurologists interpreting cognition, MRI, and plasma biomarkers in CKD is provided in Table 2.

**Table 2:** Practical checklist for interpreting cognition, MRI, and plasma biomarkers in CKD

Clinical domain	What to document	Why it matters for neurologists	Interpretation and limitations	Key references
Kidney phenotype and systemic risk	CKD stage; eGFR and/or cystatin C; albuminuria/proteinuria; diabetes/DKD; anemia; inflammation; vascular burden.	Defines the systemic context in which cognition, MRI injury, and biomarker concentrations are interpreted.	eGFR is not a direct brain-clearance measure; CKD phenotypes differ and should not be collapsed into a single “uremia” label.	[1–5, 23–28, 40, 47, 48]
Cognitive phenotype	MoCA/MMSE plus domain-specific testing when possible: executive function, processing speed, attention, episodic memory, language, and visuospatial cognition.	CKD-related impairment often has executive/attention/processing-speed features but may overlap with vascular cognitive impairment or AD-like presentations.	Screening tests alone are insufficient for mechanistic attribution; domain-level testing is preferable in studies and memory-clinic evaluation.	[1–3, 23–28, 46]
Conventional MRI and vascular brain injury	WMH burden; lacunes; microbleeds; cortical/hippocampal atrophy; PVS; global CSVD burden; prior TIA/stroke.	CKD is linked to CSVD and mixed vascular-neurodegenerative phenotypes, which may explain part of the cognitive profile.	CSVD burden does not exclude a clearance phenotype; vascular, uremic, and neurofluid mechanisms can coexist in the same patient.	[1, 2, 27, 35, 46]
Neurofluid and glymphatic imaging	When available: CPV, PVS burden, DTI-ALPS/ALPS index, gBOLD-CSF coupling, NVC metrics, ASL/CBF coupling.	Supports research-level characterization of an integrated barrier, vascular, white-matter, and neurofluid tissue phenotype	These are research markers rather than clinical diagnostic tests. DTI-ALPS is a ratio-based, structure-dependent diffusion marker whose value may be altered by numerator or denominator changes, white-matter microstructure, extracellular water, acquisition factors, and dialysis-related fluid shifts; CKD-specific diagnostic thresholds have not been validated.	[9–17, 41–46]
Dialysis and sampling context	Dialysis modality; vintage; timing of MRI/blood sampling relative to HD/PD; ultrafiltration volume; intradialytic hypotension; albumin; hemoglobin.	Dialysis-related hemodynamic and osmotic shifts may modify cerebral perfusion, CSF dynamics, neurovascular coupling, and biomarker concentrations.	Available studies have not demonstrated consistent short-term normalization of DTI-ALPS after dialysis initiation or a single HD session; modality effects remain incompletely resolved, and treatment-related changes should not be overinterpreted.	[10–12, 15, 23–28, 44, 45]
AD and neurodegeneration biomarkers	p-tau181, p-tau217, Aβ42/40, NfL, GFAP when measured; report eGFR/cystatin C and report absolute concentrations and ratios separately; use kidney-adjusted models where validated. Ratios may attenuate shared kidney-function effects but should not be considered uniformly independent of eGFR or assay platform.	Reduced kidney function may increase circulating biomarkers independently of central AD pathology, creating diagnostic vulnerability.	Avoid diagnosing AD from plasma positivity alone in advanced CKD; interpret with kidney function and confirm with CSF, amyloid/tau PET, or validated adjusted algorithms when clinically decisive.	[23–28, 47, 48]
Integrated neurologic classification	Classify the case as predominantly vascular, uremic/toxic, clearance/neurofluid, neurodegenerative, or mixed; standardize repeat testing conditions.	Creates a practical bridge between nephrology variables and neurologic interpretation in cognitive clinics, stroke clinics, and biomarker studies.	This framework is hypothesis-generating and should guide structured interpretation, not replace standard dementia, stroke, or CKD guidelines.	[1–5, 9–17, 23–28, 40–48]

Abbreviations: *Aβ42/40* amyloid-β42/amyloid-β40 ratio, *AD* Alzheimer disease, *ALPS* analysis along the perivascular space, *Aβ* amyloid-β, *ASL* arterial spin labeling, *CBF* cerebral blood flow, *CKD* chronic kidney disease, *CPV* choroid plexus volume, *CSF* cerebrospinal fluid, *CSVD* cerebral small vessel disease, *DKD* diabetic kidney disease, *DTI-ALPS* diffusion tensor imaging analysis along the perivascular space, *eGFR* estimated glomerular filtration rate, *GFAP* glial fibrillary acidic protein, *gBOLD–CSF* global blood oxygen level-dependent–cerebrospinal fluid coupling, *HD* hemodialysis, *MMSE* Mini-Mental State Examination, *MoCA* Montreal Cognitive Assessment, *MRI* magnetic resonance imaging, *NfL* neurofilament light chain, *NVC* neurovascular coupling, *PD* peritoneal dialysis, *PET* positron emission tomography, *PVS* perivascular spaces *p-tau* phosphorylated tau, *TIA* transient ischemic attack, *WMH* white-matter hyperintensities

Note: This checklist is intended for structured neurologic interpretation and research reporting; it does not define diagnostic thresholds or replace formal dementia, stroke, or CKD guidelines

Answering these questions requires intentional capture of variables that are often omitted from routine assessments. We suggest that future studies and specialized practice systematically record CKD stage, eGFR or cystatin C, dialysis modality and dialysis vintage, timing of MRI relative to the dialysis session, ultrafiltration volume, serum albumin, inflammatory markers, anemia status, sleep disturbance, overall vascular burden, WMH, cerebral microbleeds, perivascular spaces (PVS), CPV, DTI-ALPS index, gBOLD-CSF coupling, and cognitive performance assessed with the MoCA or domain-specific testing. This degree of granularity is clinically meaningful: a lower DTI-ALPS index, reflecting altered localized directional diffusivity rather than directly measured glymphatic dysfunction, has been observed in early predialysis CKD [13, 16, 17]. In contrast, one longitudinal study found no statistically significant change in the index three months after dialysis initiation [11]. These findings reinforce the need to standardize the timing of imaging in relation to dialysis and volume status, but they do not demonstrate that the same patient necessarily undergoes a treatment-related change in brain-clearance physiology (Table 3).

**Table 3:** Interpretive taxonomy of structural, diffusion-derived, CSF-dynamic, neurovascular, and blood biomarkers in CKD

Marker category	Representative markers	Primary domain sampled	Appropriate inference in CKD	What must not be inferred / key reporting requirements	Key references
Structural markers	CPV; PVS burden; WMH; lacunes; microbleeds; cortical and hippocampal volumes.	Macroscopic anatomy, cerebral small-vessel injury, and remodeling of the blood-CSF interface.	Defines structural burden and provides anatomical context for diffusion-derived, CSF-related, and neurovascular findings. CPV may serve as a candidate marker of choroid plexus remodeling.	CPV is morphologically nonspecific and cannot distinguish epithelial hypertrophy, fibrosis, edema, permeability or vascular changes, aging effects, or ventricular geometry. PVS and WMH do not directly measure CSF flow, solute transport, or brain clearance. Adjust for age, intracranial/ventricular volume, and vascular risk.	[12, 20, 21, 27, 33–36, 46]
Diffusion-derived markers	DTI-ALPS index and component diffusivities; free-water measures; DTI_low-b and periarterial FA.	Localized directional water diffusivity, white-matter microstructure, extracellular geometry, and the perivascular tissue environment.	Supports characterization of a structure-dependent brain tissue-environment phenotype. The ALPS ratio and, when available, its numerator and denominator components should be interpreted together.	DTI-ALPS is not tracer-based and does not directly quantify glymphatic flow. A lower ratio may reflect a lower numerator, a higher denominator, or both. Results are influenced by fiber orientation/crossing, white-matter injury, free water, vascular geometry, head position, ROI placement, acquisition and preprocessing, volume/osmolality, and dialysis-related fluid shifts. No CKD-specific diagnostic threshold is validated.	[10–17, 40–42]
CSF-dynamic and neurofluid markers	gBOLD-CSF coupling; validated CSF-flow or CSF-signal measures when available.	Temporal coupling between global cerebral hemodynamic fluctuations and ventricular CSF signals; macroscopic CSF-related dynamics.	Provides a complementary window on neurofluid physiology that is distinct from structural volumetry and localized diffusion indices.	Reduced coupling does not directly demonstrate solute clearance failure or glymphatic dysfunction. It may be influenced by cardiac and respiratory cycles, vigilance/sleep state, head motion, vascular reactivity, acquisition design, and signal-processing choices. Current CKD evidence is based on small cross-sectional cohorts without clinical thresholds.	[29, 30, 43, 44]
Neurovascular markers	ALFF-CBF coupling; ASL perfusion; regional and global NVC metrics.	Relationship among neural activity, cerebral perfusion, vascular responsiveness, and pulsatility.	Identifies neurovascular dysregulation that may coexist with cognitive impairment and altered structural or diffusion-derived markers in CKD.	Associations and cross-sectional mediation models do not establish temporal ordering or biological mediation. Interpret with blood pressure, anemia, carbon dioxide, diabetes, vascular burden, medications, dialysis timing, ultrafiltration, and volume status.	[9, 44–46]
Blood biomarkers	p-tau181; p-tau217; Aβ40; Aβ42; Aβ42/Aβ40 and p-tau217/Aβ42 ratios; NfL; GFAP.	Peripheral analyte concentrations jointly shaped by central release/pathology, systemic distribution and metabolism, and kidney-dependent elimination.	May support assessment of AD-related or neuroaxonal pathology only when integrated with contemporaneous eGFR and/or cystatin C, the clinical phenotype, and confirmatory testing when clinically decisive. Ratios may be more robust than isolated concentrations.	Lower kidney function may increase absolute concentrations independently of central AD pathology. Ratios may attenuate, but do not uniformly eliminate, kidney-related confounding and remain assay- and CKD-stage-specific. Advanced CKD and kidney failure are underrepresented in validation cohorts; plasma positivity alone should not establish AD in these populations.	[23–28, 47, 48]

Abbreviations: *AD* Alzheimer disease, *ALFF-CBF* amplitude of low-frequency fluctuation-cerebral blood flow coupling, *ALPS* analysis along the perivascular space, *Aβ* amyloid-β, *Aβ40* amyloid-β40, *Aβ42* amyloid-β42, *ASL* arterial spin labeling, *CBF* cerebral blood flow, *CKD* chronic kidney disease, *CPV* choroid plexus volume, *CSF* cerebrospinal fluid, *DTI-ALPS* diffusion tensor image analysis along the perivascular space, *DTI_low-b* low b-value diffusion tensor imaging with prolonged echo time, *eGFR* estimated glomerular filtration rate, *FA* fractional anisotropy, *GFAP* glial fibrillary acidic protein, *gBOLD–CSF* global blood oxygen level-dependent–cerebrospinal fluid coupling, *MRI* magnetic resonance imaging, *NfL* neurofilament light chain, *NVC* neurovascular coupling, *PVS* perivascular spaces, *p-tau* phosphorylated tau, *ROI* region of interest, *WMH* white-matter hyperintensities

Note: Categories are interpretive rather than mechanistically exclusive. No single marker establishes glymphatic flow, whole-brain clearance, temporal ordering, causal mediation, or a CKD-specific neurological diagnosis. Multimodal convergence may strengthen phenotyping but does not establish causality. DTI-ALPS should be interpreted as a structure-dependent composite diffusion marker; CPV as a morphologically nonspecific structural marker; and plasma biomarkers with contemporaneous kidney-function measures, separate reporting of absolute concentrations and ratios, and assay-specific thresholds

Diabetic kidney disease was associated with lower DTI-ALPS indices and cognitive performance in nondialysis-dependent ESRD [40]. In separate cohorts, greater CPV and lower DTI-ALPS indices were associated with verbal memory recognition [12], while CSF-related imaging abnormalities co-occurred with neurovascular decoupling in dialysis patients with mild cognitive impairment [44]. This is not proposed as a rigid rule, but as a research-informed kidney–brain clearance framework designed to standardize what is measured, so that vascular, uremic, neurovascular, and clearance-related contributions can be differentiated rather than grouped together.

Cystatin C is particularly relevant in older cognitive populations because sarcopenia, frailty, malnutrition, reduced physical activity, and neurological disability may reduce creatinine generation and cause creatinine-based eGFR to overestimate true kidney function [47, 48]. Cystatin C is less dependent on muscle mass, although it is not a pure filtration marker and may be influenced by inflammation, adiposity, thyroid dysfunction, smoking, diabetes, high cell-turnover states, and corticosteroid exposure [47, 48]. When greater accuracy is required, Kidney Disease: Improving Global Outcomes (KDIGO) recommends combined creatinine–cystatin C eGFR when available; cystatin C–based eGFR may be particularly informative when markedly reduced muscle mass is the dominant source of creatinine error and major nonfiltration determinants of cystatin C are absent [48].

## Research priorities and future directions

Despite the rapid growth of evidence associating CKD with CP remodeling, glymphatic-related imaging alterations, and cognitive decline, important methodological limitations still restrict causal interpretation of these findings. Much of the available literature is based on small, frequently single-center samples and cross-sectional designs, limiting assessment of temporal progression, causal direction, and reverse causality [2, 11–15, 40–46]. In addition, the apparent consistency of the neuroimaging literature partly reflects a limited number of centers, research groups, acquisition protocols, and post-processing pipelines, underscoring the need for multicenter independent replication [11–15, 40–46]. In addition, there is substantial heterogeneity in the CKD stages included, dialysis modalities evaluated, and criteria used to characterize cognitive impairment [11–15, 40–46]. Another relevant challenge is the inconsistent use of neuropsychological batteries across studies, which are often limited to global screening instruments such as the Mini-Mental State Examination (MMSE) and MoCA, without in-depth assessment of specific domains [11–15, 40–46]. Furthermore, few studies control for timing of data acquisition relative to HD sessions, a potentially critical factor given the acute oscillations in volume status, osmolality, extracellular water, cerebral perfusion, and CSF dynamics induced by treatment [10, 11, 15, 44, 45]. This is especially important for diffusion-derived markers such as DTI-ALPS, which may be influenced by free-water and fluid-shift effects independently of any neurofluid-related transport component [10, 16, 17]. Finally, the absence of integrated protocols combining structural and functional markers—including CPV, DTI-ALPS index, CSF dynamics, cerebrovascular imaging, PVS, white-matter lesions, and blood biomarkers—limits the construction of comprehensive pathophysiological models capable of explaining the interaction among kidney, brain, and brain-clearance systems.

Future studies should prospectively distinguish four levels of inference: association, temporal precedence, statistical mediation, and causal mediation. Cross-sectional correlations and mediation models can identify compatible relationships but cannot establish that declining kidney function precedes CP remodeling, that CP remodeling precedes changes in neurofluid markers, or that either process causes cognitive decline. Stronger inference will require repeated within-person measurements, prespecified temporal models, comprehensive adjustment for shared vascular and metabolic causes, negative-control analyses, multicenter replication, and intervention or quasi-experimental designs.

Future progress in the field will depend on the implementation of prospective multimodal designs capable of integrating neuroimaging, biomarkers, and longitudinal cognitive assessment. Cohorts of patients with CKD stages 3–5 followed over time could clarify the temporal sequence among declining kidney function, CP remodeling, impaired CSF circulation, and cognitive deterioration. MRI protocols performed immediately before and after dialysis sessions would allow characterization of the acute effects of ultrafiltration on NVC, CSF-related signal coupling, DTI-ALPS measures, and other neurofluid-related imaging markers. Comparative studies of PD, conventional HD, and hemodiafiltration (HDF) may identify kidney replacement strategies associated with lower neurobiological burden. Similarly, pre- and post-kidney transplant investigations represent a particularly valuable quasi-experimental model to determine the reversibility of uremia-associated brain alterations.

A falsifiable prediction of the framework is that interventions or major physiological transitions affecting kidney function or volume status may be followed by within-person changes in one or more CP, diffusion-derived, CSF-related, neurovascular, or cognitive measures beyond changes explained by vascular risk. Failure of a single marker to change would not validate or invalidate the entire framework because each marker samples a different and partly nonspecific component of the brain tissue environment. The absence of a significant DTI-ALPS change after dialysis initiation neither confirms nor refutes the framework [11]. The ALPS index may reflect persistent white-matter architecture, extracellular geometry, vascular factors, and fluid-related effects in addition to any neurofluid component [10, 16, 17], while the three-month observation period may be insufficient to capture structural reversibility [11]. Kidney-transplantation studies may offer a useful quasi-experimental model but will remain confounded by concurrent changes in blood pressure, anemia, inflammation, medications, nutrition, and vascular physiology.

In the future, integrated approaches combining CPV, DTI-ALPS, PVS burden, WMH, gBOLD-CSF coupling, and plasma biomarkers related to AD—including the amyloid-β 42/40 ratio (Aβ42/40), p-tau181, p-tau217, NfL, and GFAP—adjusted for eGFR and cystatin C may provide a more accurate characterization of neurodegeneration in CKD. In parallel, comprehensive neuropsychological assessments should replace exclusive reliance on screening instruments, covering episodic memory, processing speed, executive function, attention, language, and visuospatial cognition.

## Conclusions

In conclusion, CKD-associated cognitive decline should be interpreted not only as a consequence of cerebrovascular injury, chronic hypoperfusion, or uremic neurotoxicity, but also through a kidney–brain clearance framework. Emerging evidence shows that CP enlargement, altered CSF-related signal coupling, lower DTI-ALPS indices, neurovascular decoupling, neuroinflammatory activation, and kidney-related plasma biomarker confounding may coexist in CKD [2, 4, 9, 12, 13, 25, 43, 44]. These findings are compatible with, but do not establish, an integrated causal clearance pathway. From this perspective, integrating CP remodeling, CSF dynamics, perivascular diffusion-related measures, glymphatic-related imaging alterations, BBB integrity, and circulating biomarkers offers a more coherent conceptual structure for understanding cognitive vulnerability in CKD. Importantly, the kidney–brain clearance framework should not be understood as a proven unidirectional cascade linking CKD, choroid plexus remodeling, altered neurofluid physiology, and cognitive decline. It is a testable brain tissue-environment model in which barrier function, vascular pulsatility, CSF–interstitial fluid exchange, white-matter microstructure, systemic solute handling, shared vascular risk, and bidirectional relationships may interact. This model may not only refine the interpretation of future mechanistic studies, but also guide the design and interpretation of more precise diagnostic and therapeutic studies for one of the most prevalent and disabling neurological complications of contemporary nephrology.

## Data Availability

Data sharing is not applicable to this article because no datasets were generated or analyzed during the current study. All sources discussed in this narrative review are available in the published literature cited in the reference list.
